# Low‑Level Environmental Heavy Metals are Associated with Obesity Among Postmenopausal Women in a Southern State

**DOI:** 10.1007/s12403-020-00381-6

**Published:** 2021-01-17

**Authors:** Shelbie Stahr, Tung‑chin Chiang, Michael A. Bauer, Gail A. Runnells, Lora J. Rogers, Huyen Vi Do, Susan A. Kadlubar, L. Joseph Su

**Affiliations:** 1 Interdisciplinary Biomedical Sciences, Department of Clinical and Translational Sciences, University of Arkansas Medical Sciences, 4301 W. Markham St. Slot #601, Little Rock, AR 72205, USA; 2 Department of Environmental and Occupational Health, Fay W. Boozman College of Public Health, University of Arkansas Medical Sciences, 4301 West Markham, # 820, Little Rock, AR 72205, USA; 3 Department of Biomedical Informatics, College of Medicine, University of Arkansas Medical Sciences, 4018 W Capitol Ave, Little Rock, AR 72205, USA; 4 Department of Epidemiology, Fay W. Boozman College of Public Health, University of Arkansas Medical Sciences, 4301 W. Markham St. # 820, Little Rock, AR 72205, USA; 5 COPH Department of Epidemiology Slot 721-21, Winthrop P. Rockefeller Cancer Institute, Fay W. Boozman College of Public Health, University of Arkansas for Medical Sciences, 4104 Outpatient Circle, Little Rock, AR 72205-7101, USA

**Keywords:** Arsenic, Cadmium, Obesity, Saliva, Menopause

## Abstract

Both arsenic and cadmium are reported to be toxic to humans. The use of saliva as a biomarker of low-level exposures to these elements has not been adequately explored, and the putative relationship between exposure and obesity is unclear. This cross-sectional study aims to investigate the relationship between salivary arsenic and cadmium concentrations and their association with obesity. Arsenic and cadmium concentrations were analyzed in human saliva samples by Inductively Coupled Plasma-Mass Spectrometry on 270 randomly selected women who participated in the Arkansas Rural Community Health Study. Multivariable logistic regression was performed to evaluate the association between heavy metal concentrations and obesity. Stratified logistic regression was performed based on menopausal status. Generalized linear models were used to evaluate weight gain velocity. Significant positive associations were observed in postmenopausal women for both arsenic (OR = 4.43, 95% CI 1.91–10.28) and cadmium (OR = 2.72, 95% CI 1.23–5.99) concentrations, as well as significant trends among tertiles (*p* < 0.01 and *p* = 0.01, respectively). No relationship with obesity was evident among premenopausal women for either metal. A dose–response relationship was observed between increasing weight gain velocity and increasing metal concentrations. At concentrations well below governmental and industrial standards for acute toxicity, significant associations between obesity and concentration of these heavy metals are evident. The rate at which individuals gain weight is affected by metal concentrations and may play a role in the rapid increase in weight in postmenopausal women. These results might explain, in part, the missing variability in the increasing obesity pandemic in certain population exposed to these environmental toxicants.

## Introduction

Hundreds of millions of dollars are spent to prevent obesity in the United States annually (Lanza et al. 2010; Kruger et al. 2004); however, obesity rates over the past 50 years continue to climb (Wang et al. 2020). It is estimated that approximately 38% of US adults are defined as obese (NCHS 2016). Factors contributing to obesity include lack of physical activity and exercise, imbalance of energy intake and expenditure, a high sedentary lifestyle, intake of high-caloric foods, stress, genetics, and health/medical conditions (Eunice Kennedy Shriver National Institute of Child Health and Human Development 2016; Lopomo et al. 2016). The National Institute of Health has concluded that the greatest contributor to weight gain is the imbalance between caloric intake and energy expenditure (Eunice Kennedy Shriver National Institute of Child Health and Human Development 2016), but recent studies demonstrate that these factors do not fully explain the obesity problem (Park et al. 2017; Lopomo et al. 2016).

As of 2019, arsenic ranks number one on the Agency for Toxic Substances and Disease Registries (ATSDR) list of hazardous substance Priority List (ATSDR 2020). This list is created by utilizing an algorithm that takes into account the toxicity, potential for human exposure, as well as the frequency of human exposure (ATSDR 2020). Exposure to high levels of arsenic can cause skin lesions, cardiovascular disease, neurological effects, diabetogenic effects, and various forms of cancer (ATSDR 2011a; IARC 2012a). The effect of arsenic exposure in low doses remains controversial in the literature (Schmidt 2014). General population exposures to arsenic are largely attributed to contaminated food, water, and air ingestion averaging between 20 and 300 µg/day (IARC 2012a). Arsenic (As) is a naturally occurring metalloid that is ubiquitous in the environment. Arkansas is among several states along the Mississippi Delta region, which reports the highest levels of arsenic in the U.S. (Smith et al. 2017). Individuals residing in the state experience chronic low-dose exposures to these metals, and the health effects are not yet clear.

While arsenic is known to have harmful health effects regardless of gender, there are some consequences that are unique to women. Numerous studies have shown that pregnant women, fetuses, and neonates all suffer adverse pregnancy outcomes when exposed to arsenic (Milton et al. 2005, 2017; Rahman et al. 2009; Farzan et al. 2013; Ronco et al. 2010). Particularly for exposed mothers, hypertensive disorders have been reported at higher rates than in the general population. Studies of pregnant women in Chile and Romania have identified associations between adverse health outcomes among mothers consumed drinking water with arsenic concentrations of 40 μg/L (Hopenhayn et al. 2006; Munoz et al. 2018; Surdu et al. 2015). Arsenic has also been identified as an endocrine disrupter in various in vivo, in utero, and cell culture models (Chatterjee and Chatterji 2010; Davey et al. 2007; Waalkes et al. 2004). These disruptions can cause adverse developmental, reproductive, neurological, and immune effects (Alvarado-Cruz et al. 2018; Ashrap et al. 2019; Chen et al. 2009; Iavicoli et al. 2009; Liu et al. 2015; White et al. 2019). One study reported that an increase in arsenic resulted in decreased levels of estradiol that presented in a dose–response manner within a rat model (Chatterjee and Chatterji 2010). The study further describes that arsenic may mimic the estrogen mechanisms that disrupt the endocrine signaling pathway and reproductive failures.

Lesser known consequences of arsenic exposure have been observed in both experimental and epidemiological studies regarding the relationship between arsenic and obesity. An in vitro study published in 2011 concluded that prolonged inorganic arsenite exposure resulted in decreased expression in glucose transporter type 4 (GLUT4) and several adipogenic genes (Xue et al. 2011). This study indicates that prolonged exposure to arsenic causes significant effects on adipocytes (Xue et al. 2011). Similarly, an in utero study published in 2016 found that mice with early life exposure to arsenite resulted in abnormal metabolism, increased body weight, as well as other cardiometabolic risk factors (Ditzel et al. 2016). Unlike these experimental studies, epidemiological studies are less consistent. High BMI was associated with a low percentage of urinary arsenic excreted as monomethylarsonic acid in women (Gomez-Rubio et al. 2011). Contrarily, in 2010 a study evaluating if arsenic was associated with body composition in reproductive-age women found that there was no association between the two after adjusting for food consumption and lifestyle factors (Ronco et al. 2010).

Cadmium (Cd) has long been recognized as an environmental risk factor for multi-organ dysfunction and has been determined to play a role in the pathogenesis of obesity, diabetes, and the metabolic syndrome (Tinkov et al. 2017). Like arsenic, cadmium is ubiquitous in the environment and is in the top ten environmental chemicals of concern to environmental health agencies (Park et al. 2017). There is an abundance of literature focusing on cadmium exposure via in utero, perinatal, and infant exposure, but limited data on the relationship between adult exposure and obesity exist (Park et al. 2017). Human studies have produced conflicting results (Tinkov et al. 2017). Data from the National Health & Nutrition Examination Survey (NHANES), reported urine cadmium concentrations were negatively associated with BMI, waist circumference, and obesity (Padilla et al. 2010). However, a study utilizing blood cadmium levels reported a positive association between cadmium and BMI, as well as cadmium and dyslipidemia (Zhou et al. 2016).

The obesity epidemic has dramatically increased, particularly in the southern region of the United States (CDC 2019), including Arkansas. Among women, Arkansas has the highest prevalence of obesity compared to all other states (America’s Health Rankings 2020). This study conducted in an Arkansas population aimed to identify other putative contributors to the growing obesity epidemic. We sought to evaluate the potential association of arsenic and cadmium with obesity among women in Arkansas, where obesity and exposure to these elements are prevalent.

## Methods

### Study Population

Data obtained from the Arkansas Rural Community Health (ARCH) Study cohort, formerly known as Spit for the Cure (Bondurant et al. 2011; Lee et al. 2014; McElfish et al. 2019) was utilized in the present study. All 75 counties in Arkansas are represented in this cohort, including an overrepresentation of African Americans. The ARCH population was not intended to be representative of the entire female population of Arkansas. In addition to being a racially diverse cohort, it is also comprised of individuals with a higher education attainment compared to the overall state average. Data were collected at baseline that contained information regarding, family and medical history, specific information regarding breast cancer history and treatment, reproductive health, physical activity, and socioeconomic status. For each participant, a baseline 2 mL self-collection saliva sample was collected at the time of study enrollment. Baseline information was collected through various community events from a total of 26,347 women between the ages of 18 and 100.

A random sample of 270 subjects (~ 1% of the ARCH cohort) was selected and concentrations for arsenic and cadmium were quantified for the present study. This study is pilot in nature to explore our hypothesis. Future studies utilizing a larger sample size from the cohort with prospective follow-up will be conducted as well as the addition of more underlying conditions. Baseline questionnaire data were used for analysis, as well as 400 µL of the baseline saliva sample.

### Sample Preparation and Quantification by ICP‑MS

Participants were asked to refrain from eating or drinking at least 30 min prior to providing a saliva sample. Participants were also discouraged from providing a sputum sample, as mucus was not desired. Roughly 200 µL of a spit sample was mixed with equal parts Oragene DNA (OG-250) stabilizing solution (DNA Genotek, Ottawa, Ontario, Canada) (Barai et al. 2017), inverted 3–4 times to ensure distribution of solution and stored in the dark at room temperature until analysis. To obtain arsenic and cadmium concentrations, samples were analyzed using a Thermo Fisher Scientific iCAP RQ ICP-MS (Thermo Fisher Scientific, Waltham, MA).

Multi-element ICP-MS stock standard and internal standards of 10 µg/mL was purchased from Inorganic Ventures, Christiansburg, VA. A second source quality control standard (100 µg/mL) was purchased from SPEX CertiPrep, Metuchen, NJ. Nitric acid trace metal grade and ethyl alcohol was purchased from Fisher Chemical, Fair Lawn, NJ. The sample diluent used to prepare the standards, quality controls, and samples consisted of 2% (v/v) nitric acid and ethanol.

Arsenic and cadmium, among other elements, are in the multi-element ICP-MS stock standard and quality control standard solutions. The internal standard used for ICP-MS analysis was Yttrium (10 µg/L). Saliva samples were homogenized by inverting several times prior to analysis. All samples were analyzed in kinetic energy discrimination (KED) mode. Blank Oragene DNA stabilizing solution without saliva was tested and confirmed no measurable trace element of interest.

To verify that all samples utilized produced valid numbers, samples were compared against the Background Equivalent Concentration (BEC) (Wilschefski and Baxter 2019; Thomas 2004; CASRN E. S. C. E. S. 2014). The BEC is used to assure that the signals used in sample quantification are not due to “signal noise” resulting from background interference (CASRN E. S. C. E. S. 2014). The literature states that utilization of the BEC is a more reliable assessment in real-world sample matrices in regards to instrument performance (Thomas 2004), due to the quantification of the background level. In the present study, three arsenic concentrations were below its respective BEC value, and one cadmium fell below its respective BEC. For purposes of analyses, these values were recorded as “0.00” due to the variable being analyzed in tertiles. When reporting the measures of central tendency, these values were excluded from the analysis. Less than 1.0% of cadmium concentrations were recorded below BEC and 1.1% of arsenic concentrations fell below their respective BEC values.

### Obesity

At baseline, participants self-reported their height (in.) and weight (lb.), to which the participants’ body mass index (BMI) was calculated with the following formula: BMI = [weight (kg)/height (m^2^)]. If a participant had a BMI of greater than or equal to 30, they were defined as obese. If the BMI was between 18.5 and less than 30, they were defined as non-obese. Individuals with unreliable data, such as outliers or data entry errors, were removed from the analysis (*n* = 4).

The weight gain velocity (WGV) was calculated with the formula below to observe potential associations in the rate of weight gain with different concentrations of arsenic and cadmium. Individuals were asked to self-report their best approximation of their weight at age 18 during the baseline survey. If there were missing records for an individual’s weight, age, or weight at age 18, their WGV was reported as missing (*n* = 1):

$$Weight\;Gain\;Velocity=\frac{\left(Weight\;at\;enrollment\;-\;Weight\;at\;age\;18\right)}{\left(Age\;at\;enrollment\;-\;8\right)}$$

### Other Covariates

Sociodemographic information provided in the baseline survey such as race, hormone use, region of residence, education, breastfeeding/parity, alcohol use, age, physical activity, and age at last menstrual cycle were included to identify confounding effects and effect modification. The variables race, hormone use, region, education, breastfeeding/parity, and alcohol use were analyzed as categorical variables, while the variables age, physical activity, and age at last menstrual cycle were analyzed as continuous variables. Physical activity was a composite variable that varied from walking to vigorous activity, resulting in total hours per week. Race was analyzed as a categorical variable, white/European American (EA), black/African American (AA), and other. In the final stratified models, other racial groups were excluded from the analysis due to insufficient sample size (*n* = 12) and race was analyzed as a dichotomous variable, EA and AA. Hormone use was analyzed as a dichotomous variable (yes/no), relating to the participants’ menstrual cycle. Region was analyzed as a dichotomous variable as either Rural or Urban, classified by the subjects’ zip code at baseline using the rural–urban commuting area (RUCA) codes. Participants’ parity and breastfeeding practices were combined to create a composite variable with the following three possible outcomes: Nulliparous and never breastfed, given birth to at least one child and never breastfed, or given birth to at least one child and breastfed. This composite was created to avoid multicollinearity being introduced into the statistical models.

### Exclusions

Participants were excluded from the study to eliminate potential bias. Individuals that recorded non-reliable or missing value for height or weight, or a record of a BMI < 18.5 or > 60 were excluded from the analysis (*n* = 11). When analyzing the adjusted models, individuals were excluded in the analysis if a covariate utilized in the specific model was recorded as missing (*n* = 14). As previously described, data obtained for the current study were obtained from the existing ARCH study cohort. The ARCH cohort was established to study the risk and effects of breast cancer. In the current study, breast cancer cases were excluded (*n* = 8), to omit any potential bias that were associated with any treatment, lifestyle, or genetic factors.

### Statistical Analyses

Differences in patient characteristics between non-obese and obese individuals were evaluated using X^2^ test for categorical variables and t-tests for continuous variables. Both arsenic and cadmium concentrations were categorized into three levels based on the overall cohort distribution; the first level belonging to individuals with the lowest third of the specific element concentration, the second level comprised of the middle 33% of the concentrations, and the highest tertile contains the highest third of the specific element concentrations. Distributions of both arsenic and cadmium were determined by evaluating the measures of central tendency.

Linear regression models were attempted to observe the relationship between arsenic and cadmium and their association to obesity, while using BMI as the continuous outcome. Due to majority of participants had very low concentrations of these heavy metals, the data were heavily skewed such that normalization could not be achieved (data not shown). However, after producing stratified models, a l og_10_ normalization was attempted and successful for postmenopausal women and two regression models examining arsenic and cadmium as continuous exposures with the outcome of obesity was performed producing odds ratios (ORs) and 95% confidence intervals (95% CI). Heavy metal data of premenopausal women were unable to be adequately normalized, and therefore linear regression was not possible at the time, this is likely due to the small sample size.

Unconditional logistic regression was used to estimate unadjusted and multivariable-adjusted ORs and 95% CIs to estimate associations between increased likelihood for obesity and each metal concentration category. Tests for trend were performed by assessing median of arsenic and cadmium concentration tertiles as ordinal variables in the logistic regression model to observe their associations with obesity. These models were created due to the low concentrations of both arsenic and cadmium, to better observe a difference between relative “low”, “medium”, and “high” values.

Potential confounding variables for the association between obesity and arsenic and cadmium concentrations were included in the multivariable logistic regression models. Confounding variables were selected based on a priori knowledge, as well as a 10% change in the beta coefficient. The most parsimonious model was used for each analysis to estimate associations with obesity and arsenic and cadmium concentrations. The following covariates were used in at least one of the models presented: age, ethnicity, menopausal status, parity/breastfeeding history, age at last menstrual cycle, and alcohol use. Stratified models were used to analyze obesity with arsenic and cadmium concentrations for both premenopausal and postmenopausal women, using two different models. Generalized linear models were used to examine the regression between WGV and various metal quartiles, and comparisons were made using the lowest quartile as a reference. WGV was analyzed utilizing arsenic and cadmium quartiles rather than tertiles due to the larger sample size allowing for further categorizations to better investigate the relationship.

Interaction was assessed between arsenic and cadmium using a multiplicative model utilizing 75% cutoff points to determine the effects of “high” and “low” concentrations of each heavy metal. The test for interaction did not produce significant results; however, high arsenic values appear to enhance the associations. Therefore, we produced separate final models for arsenic and cadmium.

Each analysis performed was two-sided with p values of α less than 0.05. All analyses were performed using SAS version 9.3.

## Results

Demographic information stratified by both obesity and menopausal status is shown in Table 1. The cohort overall was split evenly between non-obese and obese women (51.9% vs. 48.1%, respectively). The study population consists of 78 women who identified as being premenopausal and 192 postmenopausal women. Among non-obese premenopausal women, the majority self-identified as European American (EA) (77.8%), whereas the majority of the obese premenopausal women self-identified as African American (AA) (59.5%). In the postmenopausal group, a large percentage of both non-obese and obese individuals identified as EA (69.2% and 60.2%, respectively). Regardless of obesity status, the majority of study participants live in rural regions of Arkansas. No premenopausal women reported using hormone therapy. Among postmenopausal women, similar percentages reported using hormones for both non-obese and obese individuals (11.5% and 11.4%, respectively). Premenopausal women differed in education status when separated by obesity. The majority of non-obese individuals attended some college or technical school, whereas obese individuals primarily completed a high school degree or GED. The majority of postmenopausal women, regardless of obesity status, completed a high school degree or GED as their highest form of education obtained. The distribution of age since menopause among postmenopausal women is rather uniform, regardless of obesity status, with roughly 30% in each category. When the variable of parity and breastfeeding were combined into one composite variable, premenopausal non-obese women mainly gave birth to children and did breastfeed (52.8%) compared to the other categories, whereas premenopausal obese women, as well as all postmenopausal women primarily gave birth to children and did not participate in breastfeeding (50.0%, 54.8%, and 48.9% respectively). The majority of women in the study, both non-obese and obese, premenopausal and postmenopausal women reported their alcohol consumption as never to once a year.

The distribution of BMI was similar among premenopausal and postmenopausal women. Among premenopausal women, the non-obese women had a mean BMI of 24 kg/m^2^, whereas obese women had a mean BMI of 38 kg/m^2^. Similarly, postmenopausal non-obese women had a BMI of 27 kg/m^2^, and obese women recorded a higher BMI of 37 kg/m^2^. Premenopausal women displayed an almost 23-lb difference between non-obese and obese self-reported weight at age 18, whereas postmenopausal women reported a smaller gap of 7.4 lb. Regardless of the menopausal group, the non-obese women reported lower weights at age 18 compared to obese women. The mean ages for each group of women ranged from 44 years of age to 59 years of age. Premenopausal women recorded more hours per week participating in physical activity compared to postmenopausal women. The age at which women underwent menarche was consistent across all groups.

Distributions of both arsenic and cadmium are detailed in Table 2. All concentrations, regardless of element, are below the safety limits set by the Centers for Disease Control and Prevention (CDC), World Health Organization (WHO), Food and Drug Administration (FDA), and the Agency for Toxic Substances and Disease Registry (ATSDR) (ATSDR 2011a, b). The mean arsenic values for both premenopausal and postmenopausal women are higher for obese women compared to non-obese women (0.028 µg/L, 0.023 µg/L vs. 0.022 µg/L, 0.020 µg/L, respectively). When cadmium concentrations were considered, mean distributions differed by menopausal status. Premenopausal women had higher mean cadmium concentrations among non-obese women compared to obese (0.026 µg/L and 0.019 µg/L, respectively). Among postmenopausal women, those that were obese had a higher mean cadmium concentration compared to those who identified as non-obese (0.110 µg/L and 0.020 µg/L, respectively). It should be noted that one individual in the study population had a significantly higher cadmium concentration that the rest of the participants; however, when excluding this participant, the relationships between obese and non-obese individuals remain (results not shown).

**Fig. 1:**
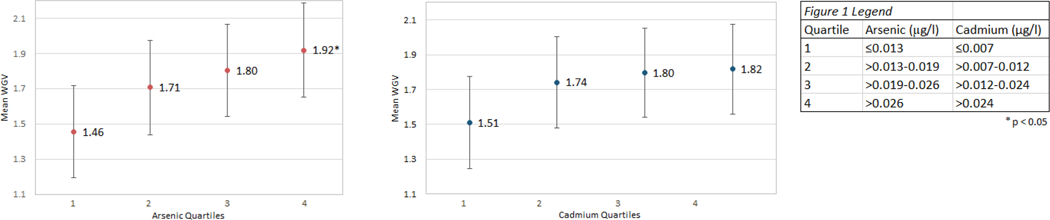
Regression between the weight gain velocity LS-means and arsenic quartiles and between the weight gain velocity LS-mean and cadmium quartiles determined using simple linear regression

Table 3 displays the associations between obesity and metal concentrations. No models comprised of premenopausal women produced significant relationships or trends regarding metal concentration and obesity. Among postmenopausal women, relationships between obese and heavy metals were evident. A significant association was observed for increasing concentrations of arsenic and risk for obesity, including a significant trend (*P* for trend < 0.01). Although the relationship with obesity was less robust, cadmium concentrations followed a similar direction. A significant increase in risk for obesity can be seen with increasing cadmium concentrations, as well as a significant trend (P for trend = 0.01). These trends suggest a dose–response for both arsenic and cadmium, and their relationship with an increased risk for obesity. Observing arsenic as a continuous variable for postmenopausal women was also produced and yielded statistically significant results similar to results of the logistic regression models. For every 10 unit increase in arsenic, the odds of being obese increase by a factor of 19.01. Similarly, for every 10 unit increase in cadmium, the odds of being obese increase by a factor of 2.64. Models were only ran among postmenopausal women, as premenopausal women did not fit a normal distribution among heavy metals. Weight gain velocity was calculated to identify potential associations between metal concentrations and the rate of weight gain since 18 years of age. Figure 1 displays the comparison of mean metal concentrations displayed in quartiles compared to their respective weight gain velocities. While only the first and fourth arsenic quartile show a statistically significant difference (*p* < 0.05), the overall positive trend can be seen for increasing metal concentration and increasing weight gain velocity for both cadmium and arsenic that suggest dose–response relationships.

## Discussion

Associations between both salivary arsenic and cadmium concentrations and obesity were examined in a pilot study using data from a cohort of Arkansan women. The associations found in this study relating to obesity were evident only among postmenopausal women. While it is known that weight gain is a common result after menopause (Al-Safi and Polotsky 2015; Lovejoy 2003), a change in fat distribution also occurs (Lovejoy 2003; Al-Safi and Polotsky 2015). While the mechanism is unclear, there are several factors that influence this distribution, such as hormone replacement therapy, age, and body composition (Lovejoy 2003). Another factor that should be recognized are estrogen levels. While there is sparse epidemiologic data regarding the relationship between estrogen and arsenic, the results produced from the current study support the existing evidence. The relationship seen between arsenic and obesity among postmenopausal women in the current study supports the relationship of a decrease in estrogens as a result of increased arsenic concentrations seen in other publications (Chatterjee and Chatterji 2010).

While these results are promising, the mechanisms of these relationships have yet to be ascertained.

Furthermore, there are several biologically plausible hypotheses of the associations between arsenic and obesity seen in this study.

Arsenic is typically measured in urine, blood, hair, and fingernails/toenails as appropriate biospecimens for exposure, while saliva is not as commonly used. Cadmium is more commonly measured in blood, urine, and kidney samples. A study published in West Bengal, India, analyzed saliva and concluded that salivary arsenic could be used as a potential biomarker of arsenic exposure, compared to urine arsenic concentrations (*r* = 0.60) (Bhowmick et al. 2013). A similar study published in *Environmental Health and Preventative Medicine* concluded that saliva might be utilized as a biological monitoring tool for arsenic, due to their correlation analysis finding significant positive correlations between total arsenic in urine and saliva (*r* = 0.794, *p* < 0.01) (Wang et al. 2017). We also found a significantly correlation in our pilot study of 100 volunteers for the concentration of arsenic between saliva and urine (*r* = 0.60, *p* < 0.01) after adjusting for age, race, sex, BMI, and smoking status (not published). Positive aspects of utilizing saliva as a biospecimen are that it is a relatively stable matrix compared to blood and urine, the processing and storage of the sample is less tedious compared to other biofluids, as well as the noninvasive procurement (Bhowmick et al. 2013; Wang et al. 2017). Limitations of the use of saliva are related to the fact that the matrix has a different chemical composition compared to blood or urine, the concentrations are considerably lower in saliva compared to traditional biological samples used (often 1 to 2 orders of magnitude lower than blood), and a lack of Standard Reference Material set by the US National Institute of Standards and Technology (Bhowmick et al. 2013; Wang et al. 2017).

The relationship between urinary arsenic concentration and BMI remains inconsistent in the exiting published reports. A study published in *Toxicology and Applied Pharmacology* in 2011 found that a decrease in urinary arsenic concentration is associated with an increase in BMI (Gomez-Rubio et al. 2011). One hypothesized explanation for this relationship is that arsenic is stored and accumulates in adipose tissue. In the current study, postmenopausal women had a significant correlation with increasing arsenic concentrations and obesity. Fat distribution in women changes with menopausal status, where central/abdominal obesity is more prevalent among postmenopausal women, as are various hormone stores within this white adipose tissue (Garaulet et al. 2002; Ferrara et al. 2002; Hodson et al. 2015; Lovejoy, 2003; Ceja-Galicia et al. 2017). The decreased urinary arsenic concentration associated with an increase in BMI could possibly be explained by the urinary quantification. With the arsenic being stored in adipose tissues and only a marginal amount being excreted in the urine, it is possible that a low amount of arsenic is associated with a high BMI. In reality, these results are due to a large concentration of arsenic present, but it is stored in the adipose tissue, thus causing a bias moving away from the null value, and could explain a number of study findings in the literature.

In contrast, fewer results published have concluded that an increase in urinary arsenic concentration is associated with a decrease in BMI (Ronco et al. 2010); however, the inverse relationship can still be explained by the same mechanism as described above. With a low BMI, there is not an abundance of adipose tissue to be utilized for arsenic storage, and therefore the majority is excreted in the urine. These results can inaccurately depict that high urinary arsenic is associated with a low BMI, where, in reality, there is no arsenic being stored in the body and is all excreted, thus biasing the results.

By utilizing saliva, these potential biases can be avoided, as saliva does not undergo the same biological processes as urine. A paper published in *Environmental Health Perspectives* in 2017 suggested that the relationship between arsenic and obesity measured in urine can have drastically different results, depending upon the urine dilution adjustment method used (Bulka et al. 2017). In the present study, we report a more accurate representation of the arsenic-BMI relationship by the use of saliva as a biospecimen with less variation due to physiological factors.

Figure 1 depicts the results from the regression analysis of weight gain velocity. The study identified a positive association between the rate of weight gain since 18 years of age and elevated concentrations of salivary arsenic and cadmium. As shown in Table 3, a clear relationship between these metals and obesity is evident. Further analysis of this relative weight gain suggests that low amounts of metal concentrations affect the rate at which weight gain occurs in the lifespan. While the current study reports concentrations of As and Cd well below the governmental guidelines, a more diverse sample size with a larger range of concentrations could identify a more robust relationship between WGV and metal concentration. It is known that rapid weight gain causes nutritional deficiencies, excess adipose tissue accumulation, and increased pressure on various organ systems. With the concentrations of metals in the current study all below 1 ppb, these low concentrations could contribute to meaningful differences in WGV, thus further investigations into these relationships are needed.

It should also be noted that the literature identifies that arsenic concentrations in saliva are considerably lower when compared to blood and urine (Wang et al. 2017; Bhowmick et al. 2013; Yuan et al. 2008), which was a major technical issue a few decades ago because the lower detection limit of Atomic Absorption Spectroscopy or earlier version of ICP-MS was at part per million (ppm) or part per billion (ppb) level. The newer version of ICP-MS, as used in this study, is capable of detecting concentrations as low as parts per trillion (ppt). This allows us to reach extremely low detection limits within our population who were exposed to trace amounts of arsenic and cadmium that previous generation of instruments could not achieve. While arsenic and cadmium produce concentrations lower in saliva compared to blood and urine, their exposures remain the same, and with this study, we wish to identify the potential use of saliva as a biomarker for various metal exposures. Additionally, another primary advantage of this study is the ability to quantify both arsenic and cadmium concentrations simultaneously, allowing the concurrent observation of multiple elements and their relationship with obesity, with independent analyses per each element of interest.

This study has several limitations. First, there are covariates that were not recorded at baseline, such as smoking status and occupation, which would preferably be included in the analyses. Smoking and the effects on heavy metal concentrations are controversial in the literature (Garhammer et al. 2004). A separate pilot study consisting of 100 individuals did not provide any significant association between arsenic and cadmium concentration with smoking status (results not shown). Different occupational exposures can be related to elevated levels of arsenic and cadmium (IARC 2012a; b), as well as the risk for obesity (Church et al. 2011). Smoking is also an established cause of increased arsenic and cadmium concentrations in the body (Pappas 2011) and is associated with various occupational exposures (Sterling and Weinkam 1990). Another concern at baseline, several essential variables, such as height, weight, and weight at age 18 were collected as self-report data. These data were collected at the same interview as the saliva sample, which is a limitation of cross-sectional studies. We are unable to confirm the temporal relationship between the arsenic and cadmium concentrations with obesity. Another restriction is that the number of subjects included in subset analyses, particularly among premenopausal subjects, was relatively low. In the current study, the significant findings were among the postmenopausal women, while the premenopausal category failed to establish any significant correlations. With the sample size of 77 participants, we are unable to produce precise results, compared to that of the postmenopausal results where the sample sizes are more than doubled. However, a study published in *Biological Trace Element Research* produced similar results. Women aged 18–35 of childbearing-age were analyzed for relationships between urinary arsenic concentrations and BMI as well as the fat mass percentage (Ronco et al. 2010). The results concluded that no significant relationships could be obtained. Therefore, while the current study has a smaller sample size for the stratification of premenopausal women, the results are consistent with the current literature.

In conclusion, the relationship between arsenic and obesity, as well as cadmium and obesity were observed in postmenopausal women, both with an evident dose–response relationship. The relationship is not evident among premenopausal women for either element. Measurement in saliva, rather than in blood or urine, produced meaningful results. While these concentrations are below the regulatory and governmental limits, they still show a significant association with obesity. The data presented in this study regarding low levels of arsenic and cadmium can inform governmental limits and guidelines regarding appropriate levels of human exposure. Information presented in the current study has revealed other possible explanations to the growing obesity epidemic. Additional research is needed to further examine the magnitude of effect arsenic and cadmium have on obesity. This study has demonstrated that saliva can be an appropriate biospecimen for various elements and should be further investigated. Future research including smoking habits to more specifically analyze metal concentrations as well as any potential confounders is necessary; ambient air pollution measurements to assess the sources of exposure to these metals is also needed.

## Figures and Tables

**Table 1:** Sociodemographic characteristics of the population by obese status and menopausal status

Characteristic	Premenopausal women				Postmenopausal women			
	Non-obese†		Obese†		Non-obese†		Obese†	
	*N* = 36	(%)	*N* = 42	(%)	*N* = 104	(%)	*N* = 88	(%)
*Race*								
AA/African American	6	(16.7)	25	(59.5)	26	(25.0)	33	(37.5)
EA/European American	28	(77.8)	17	(40.5)	72	(69.2)	53	(60.2)
Missing	2	(5.5)	0	(0.0)	6	(5.8)	2	(2.3)
*Region*								
Rural	36	(100.0)	37	(88.1)	99	(95.2)	84	(95.5)
Urban	0	(0.0)	5	(11.9)	4	(3.8)	3	(3.4)
Missing	0	(0.0)	0	(0.0)	1	(1.0)	1	(1.1)
*Hormone use*								
Yes	0	(0.0)	0	(0.0)	12	(11.5)	10	(11.4)
No	36	(100.0)	42	(100.0)	92	(88.5)	78	(88.6)
Missing	0	(0.0)	0	(0.0)	0	(0.0)	0	(0.0)
*Education*								
Less than high school	2	(5.6)	3	(7.1)	16	(15.4)	14	(15.9)
High school graduate or GED	11	(30.5)	16	(38.1)	42	(40.4)	35	(39.8)
Some college or technical school	14	(38.9)	15	(35.7)	26	(25.0)	30	(34.1)
College or post graduate	9	(25.0)	8	(19.1)	20	(19.2)	9	(10.2)
Missing	0	(0.0)	0	(0.0)	0	(0.0)	0	(0.0)
*Menopausal status*								
Premenopausal	36	(100.0)	42	(100.0)	0	(0.0)	0	(0.0)
Postmenopausal for ≤ 10 years	0	(0.0)	0	(0.0)	34	(32.7)	29	(33.0)
Postmenopausal for 10–21 years	0	(0.0)	0	(0.0)	33	(31.7)	34	(38.6)
Postmenopausal for > 21 years	0	(0.0)	0	(0.0)	37	(35.6)	25	(28.4)
Missing	0	(0.0)	0	(0.0)	0	(0.0)	0	(0.0)
*Children and breastfeeding*								
Not given birth and not breastfeed	2	(5.5)	5	(11.9)	8	(7.7)	12	(13.6)
Given birth and not breastfed	15	(41.7)	21	(50.0)	57	(54.8)	43	(48.9)
Given birth and breastfed	19	(52.8)	16	(38.1)	38	(36.5)	30	(34.1)
Missing	0	(0.0)	0	(0.0)	1	(1.0)	3	(3.4)
*Alcohol use*								
Never to once a year	26	(72.2)	26	(61.9)	78	(75.0)	77	(87.5)
Once a month	5	(13.9)	11	(26.2)	10	(9.6)	3	(3.4)
Once a week to several times a week	4	(11.1)	4	(9.5)	14	(13.5)	5	(5.7)
Every day	1	(2.8)	1	(2.4)	2	(1.9)	2	(2.3)
Missing	0	(0.0)	0	(0.0)	0	(0.0)	1	(1.1)
	Mean‡	(SD)	Mean‡	(SD)	Mean‡	(SD)	Mean‡	(SD)
BMI	24	(3.0)	38	(6.0)	27	(2.7)	37	(4.8)
Missing	0		0		0		0	
Weight at 18 years of age (lbs.)	117.4	(16.5)	140.3	(38.8)	122.2	(26.1)	129.6	(29.3)
Missing	0		0		2		0	
Baseline Age (yrs.)	44	(6.7)	46	(5.2)	59	(8.8)	58	(8.1)
Missing	0		0		0		0	
Physical Activity (hrs. per week)	17.5	(1207.3)	20.5	(1590.7)	15.3	(1290.6)	12.1	(874.3)
Missing	0		0		0		0	
Age at first menstrual cycle	13	(2.0)	13	(2.0)	13	(1.8)	12	(1.8)
Missing	0		1		0		0	

*SD* standard deviation, *BMI* Body mass index, *lbs.* Pounds, *hrs.* Hours, *yrs.* Years

† Chi-square test for categorical variables

‡ T-test for continuous variables

p values calculated excluding the missing values

**Table 2:** Descriptive statistics by element concentration, obese status, and menopausal status

Arsenic	Premenopausal women						Postmenopausal women					
	N	Mean	Median	SD	Min	Max	N	Mean	Median	SD	Min	Max
Obese	41	0.028	0.017	0.044	0.008	0.261	88	0.023	0.022	0.012	0.008	0.086
Non-obese	35	0.022	0.019	0.011	0.007	0.048	103	0.020	0.017	0.012	0.007	0.090
Cadmium												
Obese	42	0.019	0.016	0.019	0.003	0.092	88	0.110	0.012	0.655	0.002	6.144
Non-obese	36	0.026	0.017	0.030	0.002	0.169	104	0.020	0.011	0.029	0.001	0.209

* Excluding concentrations that were below BEC

** Mean, Median, Min. and Max. are shown in µg/L

**Table 3:** Odds Ratios and 95% Confidence Intervals for association between element concentrations and obese status

Premenopausal women					Postmenopausal women				
Element concentrations	Non-obese	Obese	OR	(95% CI)	Element concentrations	Non-obese	Obese	OR	(95% CI)
Arsenic^a^	(*N* = 34)	(*N* = 42)			Arsenic^b^	(*N* = 97)	(*N* = 83)		
≤ 0.014 (μg/L)	11	15	1.00	(ref.)	≤ 0.015 (μg/L)	42	18	1.00	(ref.)
> 0.014—≤ 0.024 (μg/L)	12	15	0.72	(0.22–2.29)	> 0.015—≤ 0.023 (μg/L)	31	29	2.74	(1.20–6.27)
> 0.024 (μg/L)	11	12	0.63	(0.18–2.15)	> 0.023 (μg/L)	24	36	4.43	(1.91–10.28)
Trend			0.44		Trend			< 0.01	
					Arsenic (continuous)^b^			19.01	(3.39–106.48)
Cadmium^a^	(*N* = 34)	(*N* = 42)			Cadmium^b^	(*N* = 97)	(*N* = 83)		
≤ 0.009 (μg/L)	11	15	1.00	(ref.)	≤ 0.008 (μg/L)	39	20	1.00	(ref.)
> 0.009—≤ 0.021 (μg/L)	11	15	0.98	(0.31–3.13)	> 0.008—≤ 0.019 (μg/L)	32	27	1.57	(0.70–3.48)
> 0.021 (μg/L)	12	12	0.70	(0.21–2.34)	> 0.019 (μg/L)	26	36	2.72	(1.23–5.99)
Trend			0.56		Trend			0.01	
					Cadmium (continuous)^b^			2.64	(1.33–5.21)

a Adjusted for: age, ethnicity, and parity/breastfeeding

b Adjusted for: age, ethnicity, alcohol use, parity/Breastfeeding, and age at first menstrual cycle
